# Polyfunctional, Proinflammatory, Tissue‐Resident Memory Phenotype and Function of Synovial Interleukin‐17A+CD8+ T Cells in Psoriatic Arthritis

**DOI:** 10.1002/art.41156

**Published:** 2020-02-04

**Authors:** Kathryn J. A. Steel, Ushani Srenathan, Michael Ridley, Lucy E. Durham, Shih‐Ying Wu, Sarah E. Ryan, Catherine D. Hughes, Estee Chan, Bruce W. Kirkham, Leonie S. Taams

**Affiliations:** ^1^ King’s College London London UK; ^2^ King’s College London Guy’s Hospital, and St. Thomas’ Hospital London UK; ^3^ Guy’s Hospital and St. Thomas’ Hospital London UK

## Abstract

**Objective:**

Genetic associations imply a role for CD8+ T cells and the interleukin‐23 (IL‐23)/IL‐17 axis in psoriatic arthritis (PsA) and other spondyloarthritides (SpA). IL‐17A+CD8+ (Tc17) T cells are enriched in the synovial fluid (SF) of patients with PsA, and IL‐17A blockade is clinically efficacious in PsA/SpA. This study was undertaken to determine the immunophenotype, molecular profile, and function of synovial Tc17 cells in order to elucidate their role in PsA/SpA pathogenesis.

**Methods:**

Peripheral blood (PB) and SF mononuclear cells were isolated from patients with PsA or other types of SpA. Cells were phenotypically, transcriptionally, and functionally analyzed by flow cytometry (n = 6–18), T cell receptor β (TCRβ) sequencing (n = 3), RNA‐Seq (n = 3), quantitative reverse transcriptase–polymerase chain reaction (n = 4), and Luminex or enzyme‐linked immunosorbent assay (n = 4–16).

**Results:**

IL‐17A+CD8+ T cells were predominantly TCRαβ+ and their frequencies were increased in the SF versus the PB of patients with established PsA (*P* < 0.0001) or other SpA (*P* = 0.0009). TCRβ sequencing showed that these cells were polyclonal in PsA (median clonality 0.08), while RNA‐Seq and deep immunophenotyping revealed that PsA synovial Tc17 cells had hallmarks of Th17 cells (*RORC*/*IL*23R/*CCR6*/*CD161*) and Tc1 cells (granzyme A/B). Synovial Tc17 cells showed a strong tissue‐resident memory T (Trm) cell signature and secreted a range of proinflammatory cytokines. We identified CXCR6 as a marker for synovial Tc17 cells, and increased levels of CXCR6 ligand CXCL16 in PsA SF (*P* = 0.0005), which may contribute to their retention in the joint.

**Conclusion:**

Our results identify synovial Tc17 cells as a polyclonal subset of Trm cells characterized by polyfunctional, proinflammatory mediator production and CXCR6 expression. The molecular signature and functional profiling of these cells may help explain how Tc17 cells can contribute to synovial inflammation and disease persistence in PsA and possibly other types of SpA.

## Introduction

Psoriatic arthritis (PsA) is part of an umbrella group of inflammatory diseases, termed spondyloarthritides (SpA), that share common patterns of joint inflammation (peripheral and axial); skin, gut, and eye manifestations; genetic components; and the absence of diagnostic autoantibodies (seronegativity). In addition to PsA, SpA includes ankylosing spondylitis (AS)/nonradiographic axial spondylitis, reactive arthritis, enteropathic arthritis, and undifferentiated SpA, with a combined prevalence of 1–2% 1.

It is increasingly recognized that the interleukin‐23 (IL‐23)/IL‐17 pathway plays a major role in PsA/SpA immunopathogenesis 2, 3. Therapies targeting IL‐17A show clinical efficacy in patients with PsA and those with AS 4, 5, while several genetic loci implicated in the IL‐17/IL‐23 axis, including *IL12B* (IL‐12p40), *IL23R*, and *TRAF3IP2* (Act1) are associated with PsA and AS susceptibility 6, 7. To date, the majority of studies have focused on identifying IL‐17A–producing CD4+ T (Th17) cells or group 3 innate lymphoid cells in the inflamed joints of patients with PsA/SpA, yet the strong association of major histocompatibility complex (MHC) class I and other CD8+ T cell/MHC class I–related loci (*RUNX3, ERAP1/2*) suggests that CD8+ T cells play an important role in PsA/SpA 7, 8, 9. We previously demonstrated the enrichment of IL‐17A–expressing CD8+ T (Tc17) cells in the synovial fluid (SF) of patients with PsA 10; Tc17 cells have also been observed in the SF of patients with juvenile idiopathic arthritis (JIA) 11 and at the site of inflammation in other immune‐mediated inflammatory diseases (for review, see refs. 2 and 12). Recent murine models and transcriptional analysis of healthy human spleen Tc17 cells have shed light on the function of Tc17 cells 13, 14. However, functional and molecular analysis of human synovial Tc17 cells is essential to elucidate the role of synovial Tc17 cells in PsA/SpA pathogenesis.

The enrichment of Tc17 in the inflamed joint raises the question of whether these cells migrate into the joint or are persistently present. Recently, a novel subset of CD8+ effector T cells enriched in tissue compartments without significant presence in the blood has been described 15. These tissue‐resident memory T (Trm) cells are characterized by expression of CD69 and CD103, defined by a core transcriptional signature 16, and have the potential to produce proinflammatory cytokines including IL‐17A, IL‐22, and interferon‐γ (IFNγ), as well as granzymes and perforin (for review, see refs. 17 and 18). In humans, Trm cells have been observed in skin, lung, gut, and brain tissue, and a recent study demonstrated the presence of CD8+ T cells with a tissue‐resident memory phenotype in the SF of patients with JIA 11. As such, Trm cells are hypothesized to contribute to the immunopathogenesis of human immune‐mediated inflammatory disease. However, if and how Tc17 and Trm cells relate to each other is not well established.

To enhance our understanding of the function and molecular biology of human IL‐17A+CD8+ T cells, we performed extensive phenotypic, molecular, and functional profiling of human Tc17 cells derived from PsA SF. Using flow cytometry, T cell receptor (TCR) sequencing, Luminex, and RNA‐Seq analysis, we demonstrated that PsA synovial IL‐17A+CD8+ T cells have a polyclonal TCR repertoire, a polyfunctional, proinflammatory cytokine profile, and many hallmarks of Trm cells. These features position Tc17 cells as relevant contributors to the initiation or perpetuation of chronic inflammation in PsA, and possibly other SpA or IL‐17A/HLA class I–associated inflammatory diseases.

## Materials and Methods

#### Study subjects

Peripheral blood (PB) and SF samples were obtained from patients with PsA or other types of peripheral SpA (AS/nonradiographic axial SpA, reactive arthritis, and enteropathic arthritis) who were seen in the Rheumatology Department of Guy's Hospital. Patients fulfilled the Classification of Psoriatic Arthritis Study Group or American College of Rheumatology/European League Against Rheumatism 2010 criteria 19, 20. The demographic and clinical characteristics of patients are shown in Supplementary Table 1, available on the *Arthritis & Rheumatology* web site at http://onlinelibrary.wiley.com/doi/10.1002/art.41156/abstract. All subjects provided written informed consent. Ethics approval was obtained from Bromley Research Ethics Committee (06/Q0705/20) and Harrow Research Ethics Committee (17/LO/1940).

#### Cell isolation

Mononuclear cells (PB mononuclear cells [PBMCs] and SF mononuclear cells [SFMCs]) were isolated using Lymphoprep (Axis‐Shield) and washed in culture medium (RPMI 1640 supplemented with 10% fetal calf serum [FCS] + 1% penicillin/streptomycin/l‐glutamine). Cells were cryopreserved and stored in liquid nitrogen in culture medium supplemented with 50% FCS and 10% dimethyl sulfoxide (all from ThermoFisher).

#### Flow cytometric analysis

Thawed cells were rested for 1 hour at 37°C in an atmosphere of 5% CO_2_. For intracellular staining, samples were stimulated with phorbol myristate acetate (PMA; 50 ng/ml) and ionomycin (750 ng/ml) (both from Sigma‐Aldrich) in the presence of GolgiStop (BD Biosciences) for 3 hours at 37°C in an atmosphere of 5% CO_2_. Cells were stained with eFluor 780 Viability Dye (eBioscience), and surface staining was performed at 4°C. Cells were fixed with 2% paraformaldehyde and permeabilized using 0.5% saponin (Sigma‐Aldrich). Antibodies are listed in Supplementary Table 2, available on the *Arthritis & Rheumatology* web site at http://onlinelibrary.wiley.com/doi/10.1002/art.41156/abstract. Samples were acquired using an LSRFortessa system (BD Biosciences). Data were analyzed using FlowJo (version 10; Tree Star).

#### TCRβ sequencing

Extracted DNA (Qiagen) was subjected to bias‐controlled amplification of V–D–J rearrangements followed by high‐throughput sequencing (immunoSEQ; Adaptive Biotech). Data from productive reads (sequence level) were analyzed using an immunoSEQ analysis platform (Adaptive Biotech). Clonality was defined as 1 – Pielou's evenness and ranged from 0 (indicating a highly polyclonal repertoire) to 1 (indicating a monoclonal repertoire). Overlap was determined using the Morisita index, with possible scores ranging from 0 (indicating no similarity between 2 populations) to 1 (indicating complete similarity between 2 populations).

#### Cell sorting

For Trm cell sorting, SFMCs were stained with eFluor 780 and CD3, CD4, CD8, CD14, CD69, and CD103 antibodies (Supplementary Table 2, available on the *Arthritis & Rheumatology* web site at http://onlinelibrary.wiley.com/doi/10.1002/art.41156/abstract). After sorting, CD8+ Trm subsets were stimulated, fixed, and permeabilized before intracellular cytokine staining for IL‐17A and IFNγ. For sorting of cytokine‐producing cells, magnetically isolated (Miltenyi Biotec) CD3+ T cells were stimulated for 1.5 hours at 37°C with PMA (50 ng/ml) and ionomycin (750 ng/ml) before staining using an IL‐17A and, where indicated, IFNγ cytokine secretion assay (Miltenyi). To identify cytokine‐producing T cell subsets, cells were counterstained with eFluor 780, and anti‐CD3, CD8, CD14, and CD4 antibodies. Cells were sorted using a BD FACSAria and acquired using an LSRFortessa system (BD Biosciences).

#### RNA sequencing and quantitative reverse transcriptase–polymerase chain reaction (qRT‐PCR)

Libraries were prepared by Genewiz and sequenced on a HiSeq 2500 platform (Illumina) at a depth of 28–40M reads. Low‐quality bases and adapters (phred score <20) were trimmed using TrimGalore!, and reads were aligned (hg38) using RNA STAR. Paired reads were quantified using featureCounts. Principal components analysis and statistical comparison of gene expression were performed using DESeq2. RNAseq data are available via GSE137510.

For qRT‐PCR, total RNA was extracted from sorted cell subsets (Qiagen). Complementary DNA (cDNA) was generated using a high‐capacity cDNA reverse transcription kit (ThermoFisher). RT‐PCR was performed using a SensiFAST SYBR kit (Bioline) and primers from Integrated DNA Technologies (listed in Supplementary Table 3, available on the *Arthritis & Rheumatology* web site at http://onlinelibrary.wiley.com/doi/10.1002/art.41156/abstract).

#### Luminex assay and CXCL16 enzyme‐linked immunosorbent assay (ELISA)

Supernatants were obtained from sorted T cell subsets cultured for 24 hours in culture media. A custom magnetic Luminex (Bio‐Techne) was analyzed on a Luminex FlexMap 3D platform. Serum and SF were analyzed using a CXCL16 ELISA (Bio‐Techne).

#### Graphics and statistical analysis

Statistical analysis and graphic illustration were performed using either GraphPad Prism (version 7) or ggplot2 (R version 3.5.2). Results are expressed as the median and interquartile range. Wilcoxon's matched pairs signed rank test or Friedman's multiple comparisons test were performed.

## Results

#### Tc17 cells are polyclonal TCRαβ+ memory cells enriched in PsA/SpA joints

**Figure 1:**
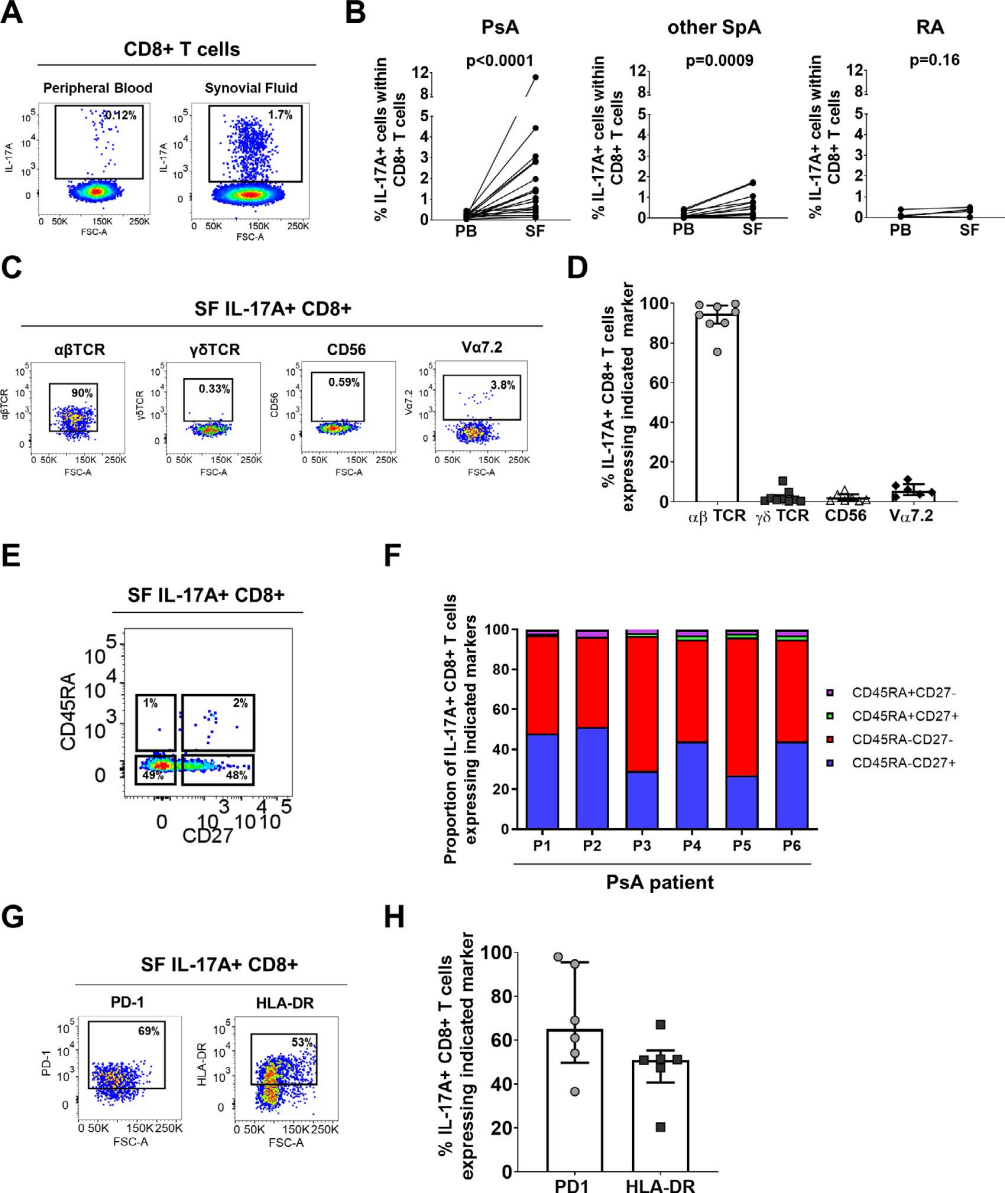
Enrichment of T cells positive for interleukin‐17A (IL‐17A) and T cell receptor αβ (TCRαβ) in the synovial fluid (SF) of patients with spondyloarthritis (SpA). **A** and **B**, Representative staining (**A**) and cumulative data (**B**) showing the frequencies of IL‐17A+ cells among CD3+CD8+ T cells in paired peripheral blood mononuclear cells (PBMCs) and SF mononuclear cells (SFMCs) from patients with psoriatic arthritis (PsA; n = 18), other peripheral SpA (n = 14), or rheumatoid arthritis (RA; n = 6) after 3 hours of stimulation in the presence of phorbol myristate acetate, ionomycin, and GolgiStop. *P* values were determined by Wilcoxon's matched pairs signed rank test. **C** and **D**, Representative staining (**C**) and cumulative data (**D**) showing the frequencies of IL‐17A+CD8+ T cells expressing TCRαβ (n = 8), TCRγδ (n = 9), CD56 (n = 7), and V_α_7.2 (n = 6) in PsA SF (stimulated as described in **A** and **B**). **E** and **F**, Representative staining (**E**) and frequencies (**F**) of IL‐17A+CD8+ T cells expressing CD45RA and/or CD27 in PsA SFMCs (n = 6) (stimulated as described in **A** and **B**). **G** and **H**, Representative staining (**G**) and cumulative data (**H**) showing the frequencies of IL‐17A+CD8+ T cells expressing programmed death 1 (PD‐1) or HLA–DR in PsA patients (n = 6). In **D** and **H**, symbols represent individual patients, bars show the median and interquartile range. Color figure can be viewed in the online issue, which is available at http://onlinelibrary.wiley.com/doi/10.1002/art.41156/abstract.

To determine whether synovial Tc17 cells are enriched only in PsA or are also enriched in other SpA types, we stimulated paired PBMCs and SFMCs from patients with PsA, patients with other types of SpA, and patients with rheumatoid arthritis (RA) ex vivo with PMA/ionomycin and assessed the frequency of IL‐17A+CD8+ T cells by flow cytometry (Figures 1A and B). (The gating strategy is shown in Supplementary Figure 1, available on the *Arthritis & Rheumatology* web site at http://onlinelibrary.wiley.com/doi/10.1002/art.41156/abstract.) Low frequencies of Tc17 cells were detected in PBMCs from patients with PsA, those with other types of SpA, and those with RA (median 0.1%), with no significant differences observed between these groups. The frequencies of Tc17 cells were significantly increased in SFMCs compared to PBMCs in patients with PsA (*P* < 0.0001) and those with other types of SpA (*P* = 0.0009), but not those with RA (*P* = 0.16). The frequencies of Th17 cells were similar between the 3 disease groups (Supplementary Figure 1B).

Synovial Tc17 cells predominantly comprised TCRαβ+ cells, with small proportions of mucosal‐associated invariant T cells (V_α_7.2+), TCRγδ, and natural killer T cells (Figures 1C and D). Over 98% of synovial Tc17 cells exhibited a memory phenotype (CD45RA−CD27+/− or CD45RA+CD27‐) (Figures 1E and F), while a considerable proportion of Tc17 cells expressed the immunoinhibitory receptor programmed death 1 (PD‐1; median 65%) and activation marker HLA–DR (median 51%), suggesting that these cells previously experienced antigen stimulation (Figures 1G and H).

**Figure 2:**
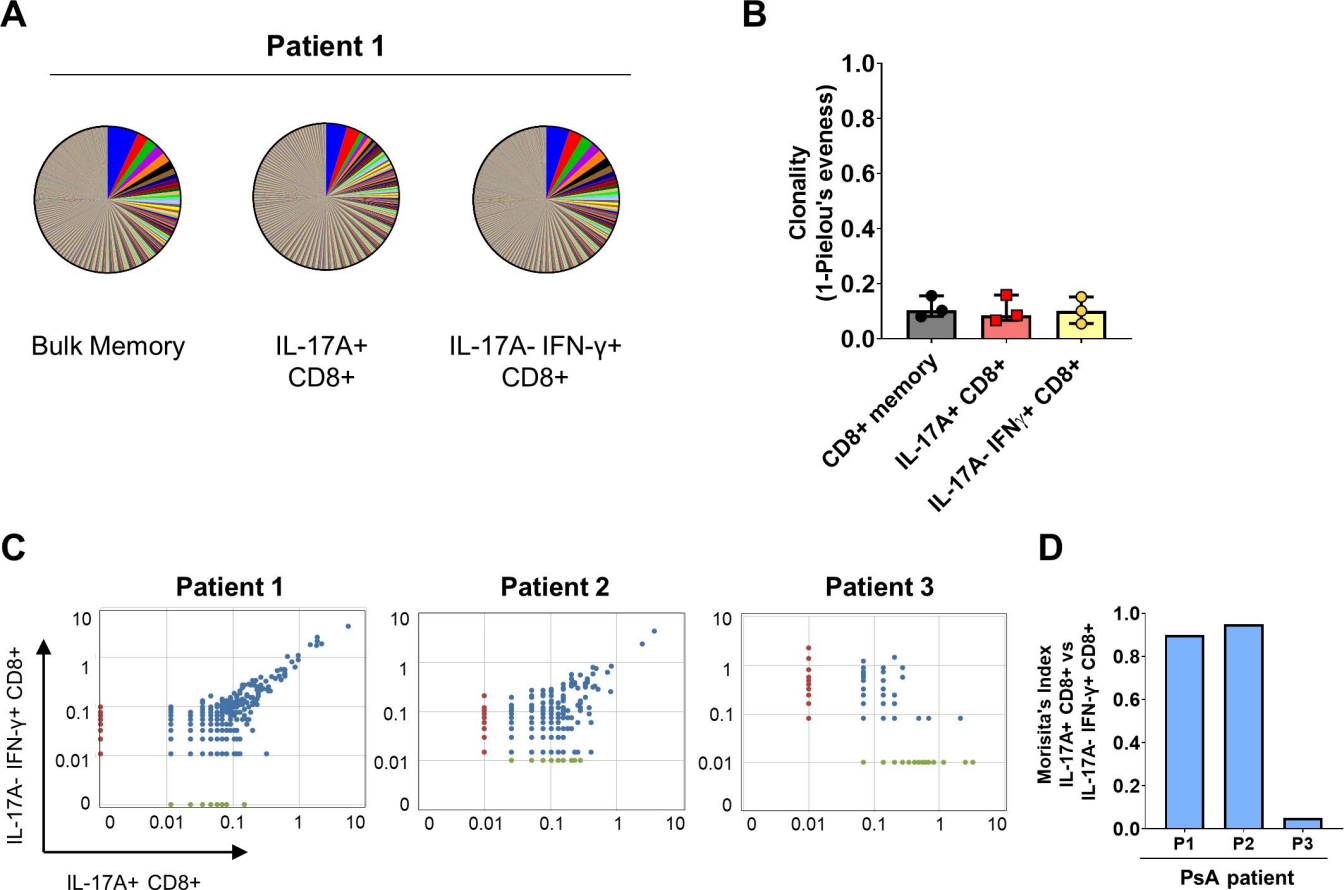
Diverse T cell receptor (TCR) repertoire in synovial Tc17 cells. **A**, Representative pie charts with segments representing the frequencies of all TCRβ sequences in bulk memory CD8+ T cells, CD8+ T cells positive for interleukin‐17A (IL‐17A), and CD8+ T cells negative for IL‐17A and positive for interferon‐γ (IFNγ) in a patient with psoriatic arthritis (PsA). **B**, Clonality score (defined as 1 − Pielou's evenness) for bulk memory CD8+, IL‐17A+CD8+, and IL‐17A−IFNγ+CD8+ T cells in PsA synovial fluid samples (n = 3). Symbols represent individual patients; bars show the median and interquartile range. **C** and **D**, Dot plots (**C**) and Morisita's index (**D**) showing the degree of clonal overlap between IL‐17A+CD8+ and IL‐17A−IFNγ+CD8+ T cells in 3 patients with PsA. Color figure can be viewed in the online issue, which is available at http://onlinelibrary.wiley.com/doi/10.1002/art.41156/abstract.

The presence of clonally restricted memory CD8+ T cells with features of antigen‐specific expansion has been described in the SF, synovial tissue, and skin of patients with PsA 21, 22, 23. To investigate whether synovial Tc17 cells are also clonally restricted in PsA, bulk memory (CD45RA−CD27+/− and CD45RA+CD27−), IL‐17A+IFNγ+/− (Tc17), and IL‐17A−IFNγ+ (Tc1) memory CD8+ T cells were sorted from the SF of PsA patients. (The gating strategy is shown in Supplementary Figure 2, available on the *Arthritis & Rheumatology* web site at http://onlinelibrary.wiley.com/doi/10.1002/art.41156/abstract.) TCRβ sequencing showed that synovial Tc17 cells have a diverse TCR repertoire (Figure 2A) with a low clonality score (Pielou's evenness), which was similar to that for synovial Tc1 and bulk memory CD8+ T cells (Figures 2A and B). For 2 patients, a substantial proportion of the clones found in synovial Tc17 cells were also present in the synovial Tc1 population, resulting in a Morisita overlap index >0.9 (Figures 2C and D). The third patient had a lower number of productive templates, which may have resulted in a low Morisita score.

#### Molecular analysis of synovial Tc17 cells reveals commonalities in transcriptional profile with Th17 and Tc1 cells

Recent transcriptional analysis of healthy human spleen Tc17 cells revealed a distinct molecular profile compared to IL‐17−CD8+ T cells or Th17 cells 14. To determine the molecular profile of Tc17 cells from the inflamed PsA joint, we sorted highly pure Tc17, Tc1, and Th17 cells from the SFMCs of patients with PsA for RNA sequencing. (The gating strategy is shown in Supplementary Figure 3, available on the *Arthritis & Rheumatology* web site at http://onlinelibrary.wiley.com/doi/10.1002/art.41156/abstract.) Accurate sorting was confirmed by the normalized gene counts of *IL17A* and *IFNG* for each of the populations (Supplementary Figure 3B).

**Figure 3:**
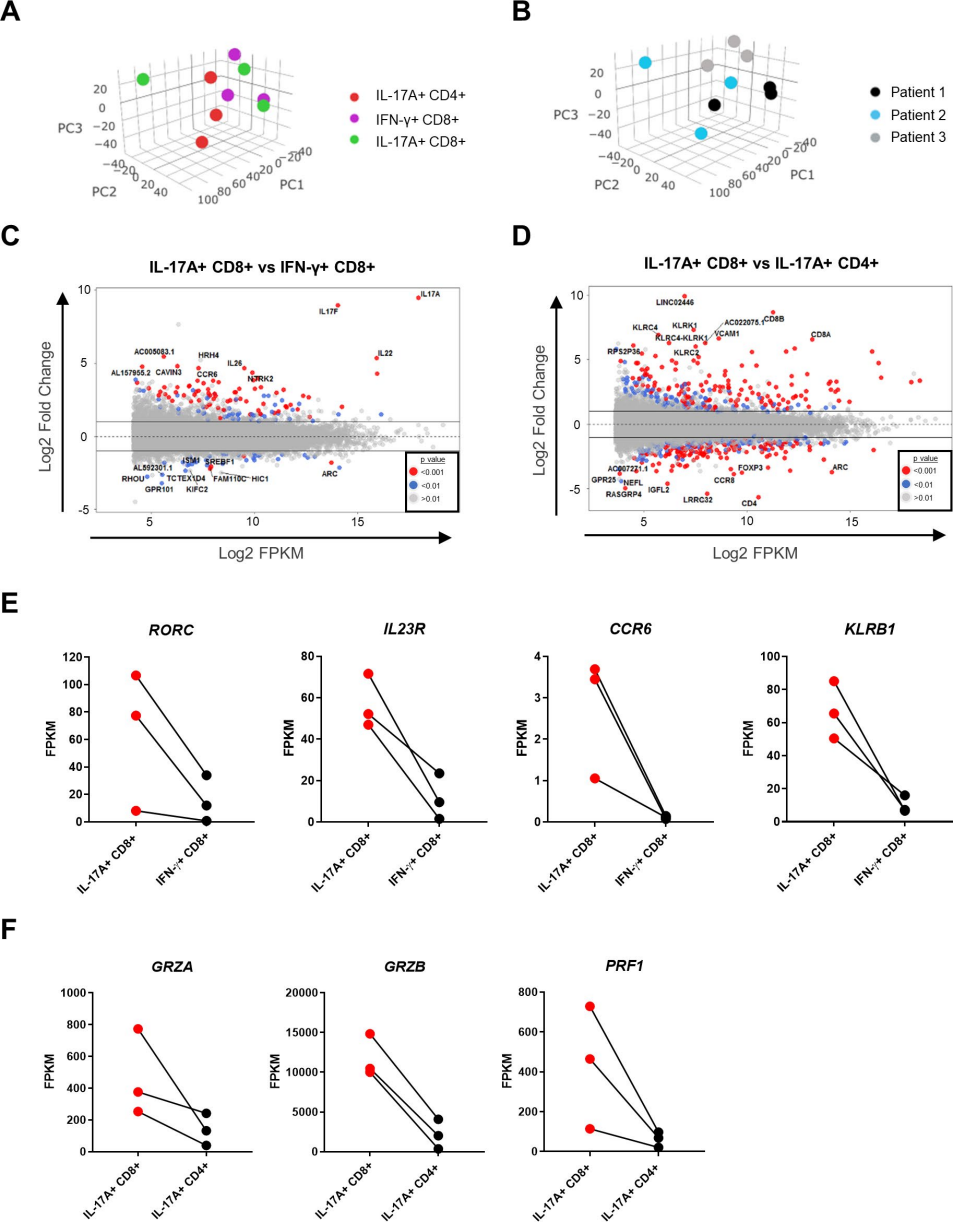
Transcriptional profile of synovial Tc17 cells compared to Tc1 cells and Th17 cells. **A** and **B**, Principal components analysis of the transcriptome of IL‐17A+CD8+, IFNγ+CD8+, and IL‐17A+CD4+ T cells from the synovial fluid of PsA patients (n = 3) by cell type (**A**) and by patient (**B**). **C** and **D**, MA plots showing genes that were significantly up‐regulated or down‐regulated in synovial IL‐17A+CD8+ T cells compared to IFNγ+CD8+ T cells or IL‐17A+CD4+ T cells (n = 3). **E** and **F**, Selected gene expression profiles shown as average gene expression values (fragments per kilobase million [FPKM]) in synovial IL‐17A+CD8+ compared to IFNγ+CD8+ T cells or CD4+IL‐17A+ T cells (n = 3). PC1 = principal component 1 (see Figure 2 for other definitions).

Principal components analysis showed a degree of gene expression heterogeneity between synovial Tc17 cells from different patients. In 2 of 3 patients, the synovial Tc17 cells clustered separately from the Th17 cells but close to the Tc1 cells (Figures 3A and B), suggesting that a proportion of the transcriptional profile is shared between synovial Tc17 and Tc1 cells. In total, 80 genes were differentially expressed between the synovial Tc17 and Tc1 subsets (≤1% false discovery rate; fold change >2). MA plots indicating the 10 genes up‐regulated to the greatest degree and the 10 genes down‐regulated to the greatest degree are shown in Figure 3C. Several genes relating to a type 17 T cell response *(IL17A, IL17F, RORC, IL23R, CCR6,* and *KLRB1)* were elevated in synovial Tc17 cells compared to synovial Tc1 cells (Figures 3C and E). When synovial Tc17 cells were compared to synovial Th17 cells, 145 genes were found to be differentially expressed (Figure 3D). Genes that were up‐regulated in synovial Tc17 cells compared to Th17 cells included *CD8A* and *CD8B*, confirming our gating strategy, plus genes associated with cytolytic activity (*GRZA, GRZB,* and *PRF1*) (Figures 3D and F). Expression levels of *TCF7* (encoding T cell factor 1), recently identified as a transcription regulator of mouse Tc17 cells through repression of MAF BZIP transcription factor and retinoic acid receptor–related orphan nuclear receptor γt 14, were low in synovial Tc17 cells (Supplementary Figure 3C).

**Figure 4:**
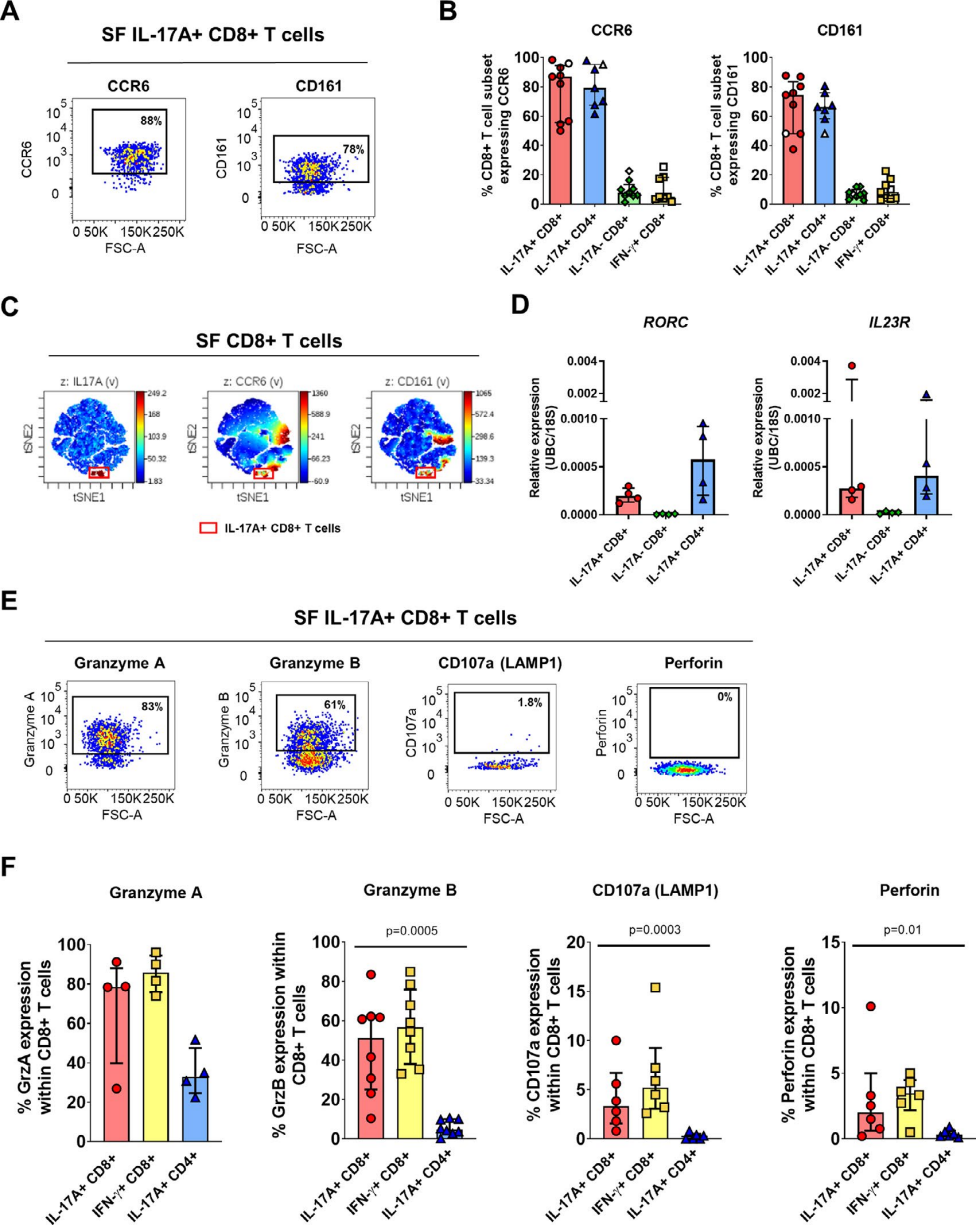
Synovial Tc17 cells have functional hallmarks of Th17 and Tc1 cells. **A** and **B**, Representative staining (**A**) and cumulative data (**B**) showing the frequencies of IL‐17A+CD8+, IL‐17A+CD4+, IL‐17A−CD8+, and IFNγ+CD8+ T cells expressing CCR6 and CD161 in synovial fluid mononuclear cells (SFMCs) from patients with PsA (solid symbols) and patients with other types of spondyloarthritis (open symbols) (n = 7–9). **C**, Representative viSNE plot showing concomitant expression of IL‐17A, CCR6, and CD161 among CD8+ T cells in PsA SFMCs. Representative results from 1 of 7 patients are shown. **D**, Gene expression levels of *RORC* and *IL23R*, normalized to the average values for *UBC* and *18S* in sorted IL‐17A+CD8+, IL‐17A−CD8+, and IL‐17A+CD4+ T cells from PsA SFMCs (n = 4). **E** and **F**, Representative staining (**E**) and cumulative data (**F**) showing the frequencies of IL‐17A+CD8+, IFNγ+CD8+, and IL‐17A+CD4+ T cells expressing granzyme A (GrzA; n = 4), granzyme B (GrzB; n = 9), CD107a/lysosomal‐associated membrane protein 1 (LAMP‐1; n = 6), and perforin (n = 6) in PsA SFMCs. *P* values were determined by Friedman's multiple comparisons test. In **B**,** D**, and **F**, symbols represent individual patients; bars show the median and interquartile range. See Figure 2 for other definitions. Color figure can be viewed in the online issue, which is available at http://onlinelibrary.wiley.com/doi/10.1002/art.41156/abstract.

We confirmed the transcription data for the type 17 T cell markers CCR6 and CD161 at the protein level by flow cytometry. A high frequency of synovial Tc17 cells coexpressed CCR6 or CD161, at levels comparable to those in synovial Th17 cells. In contrast, only limited proportions of synovial IL‐17A−CD8+ or IFNγ+CD8+ T cells expressed these molecules (Figures 4A and B). A viSNE analysis showed that CCR6 and CD161 were coexpressed by Tc17 cells (Figure 4C) (results are representative of those for 7 patients). Notably, this analysis also revealed that expression of CCR6 and CD161 was not restricted to the IL‐17A+ population, i.e., these markers do not exclusively define IL‐17A expression in synovial CD8+ T cells. Quantitative RT‐PCR analysis further confirmed that transcript levels of *RORC* and *IL23R* in the synovial Tc17 population were comparable to those in Th17 cells, and higher than those in IL‐17A−CD8+ T cells (which contains IFNγ+CD8+ T cells) (Figure 4D).

Frequencies of Tc17 cells expressing granzyme A, granzyme B, CD107a, or perforin were variable but enhanced compared to synovial Th17 cells, while they were comparable to those among synovial Tc1 cells (Figures 4E and F). It should be noted, however, that limited expression of perforin and lysosomal‐associated membrane protein 1 (LAMP‐1)/CD107a was observed in synovial Tc17 cells, suggesting that these cells may not have full cytotoxic capability.

#### Tc17 cells have hallmarks of Trm cells

**Figure 5:**
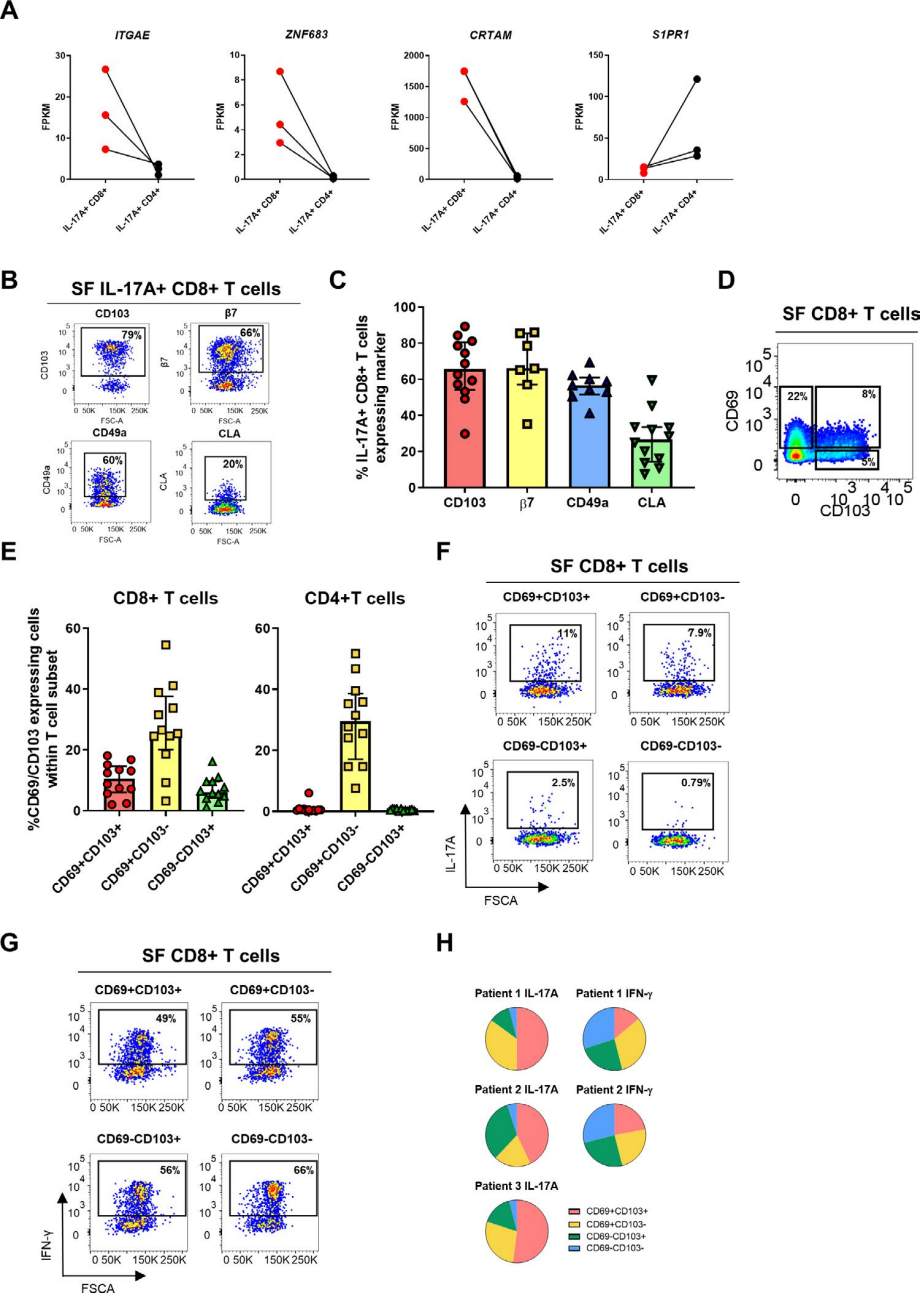
Synovial Tc17 cells form part of the synovial tissue‐resident T cell compartment. **A**, Selected tissue‐resident memory T (Trm) cell–associated gene expression profiles of sorted synovial IL‐17A+CD8+ T cells compared to IL‐17A+CD4+ T cells (n = 3). **B** and **C**, Representative staining (**B**) and cumulative data (**C**) showing the frequencies of IL‐17A+CD8+ T cells expressing CD103/αE integrin (n = 12), β7 integrin (n = 7), CD49a/very late activation antigen 1 (n = 9), or cutaneous lymphocyte antigen (CLA; n = 12) in PsA synovial fluid mononuclear cells (SFMCs), after 3 hours of stimulation in the presence of phorbol myristate acetate (PMA), ionomycin, and GolgiStop. **D** and **E**, Representative staining (**D**) and cumulative data (**E**) showing the frequencies of CD69+CD103+, CD69+CD103−, and CD69−CD103+ Trm cells among synovial CD3+CD8+ and CD3+CD4+ T cells (n = 12). **F–H**, PsA SF cells (n = 3) were sorted into CD69+CD103+, CD69+CD103−, CD69−CD103+, and CD69−CD103− CD8+ T cells. Sorted subsets were stimulated with PMA and ionomycin in the presence of GolgiStop for 3 hours. **F** and **G**, Representative staining showing IL‐17A (**F**) and IFNγ (**G**) expression within the 4 sorted subsets. **H**, Proportion of IL‐17A+ cells (left; n = 3) and IFNγ+ cells (right; n = 2) in each Trm subset, as a fraction of total IL‐17A–expressing or IFNγ‐expressing cells. In **C** and **E**, symbols represent individual patients; bars show the median and interquartile range. FPKM = fragments per kilobase million (see Figure 2 for other definitions). Color figure can be viewed in the online issue, which is available at http://onlinelibrary.wiley.com/doi/10.1002/art.41156/abstract.

The observation that Tc17 cells with a memory phenotype are enriched in the joint, but not the blood, in PsA patients prompted us to investigate whether synovial Tc17 cells from the inflamed joint expressed markers of tissue residency. Molecular profiling revealed that genes reported to be transcriptional hallmarks of Trm cells (e.g., *ITGAE* [encoding CD103], *ZNF683* [encoding HOBIT], *CRTAM*, and low levels of *S1PR1*) 16 were differentially expressed between synovial Tc17 and Th17 cells (Figure 5A). We confirmed the expression of the Trm cell marker CD103 (αE integrin) on a high proporion of joint‐derived Tc17 cells by flow cytometry (Figures 5B and C). CD69, which is also commonly used as a Trm marker, is up‐regulated upon PMA/ionomycin stimulation and was therefore not evaluated. Since Tc17 cells have been identified in the gut and the skin 24, 25, 26, 27, sites that can also be affected in PsA/SpA, we assessed markers for the gut‐related molecule β7 integrin (which is normally coexpressed with αE integrin) and the skin‐related adhesion/homing molecules cutaneous lymphocyte antigen (CLA) and CD49a (very late activation antigen [VLA‐1]). Considerable proportions of synovial Tc17 cells coexpressed β7 integrin, CD49a/VLA‐1, and to a lesser extent CLA (Figures 5B and C).

Based on these findings, we examined the presence of Trm cells using the markers CD69 and/or CD103 in unstimulated SFMCs from PsA patients (Figures 5D and E). Within the CD8+ T cell population, on average 11% of cells were CD69+CD103+ (range 2–16%), 26% of cells were CD69+CD103− (range 3.2–54%), and 6% of cells were CD69−CD103+ (range 1.29–16%). In the CD4+ compartment, 26% of the cells were CD69+CD103−, while CD103 expression was negligible (expressed by <1% of CD4+ T cells). These data indicate that CD103+ Trm cells in the inflamed PsA joint are typically CD8+ T cells.

To directly demonstrate that CD8+ Trm cells contain IL‐17A–producing cells, we sorted synovial CD8+ T cells from PsA patients into highly pure CD69+CD103+, CD69+CD103−, CD69−CD103+, and CD69−CD103− subsets. (The gating strategy is shown in Supplementary Figure 4, available on the *Arthritis & Rheumatology* web site at http://onlinelibrary.wiley.com/doi/10.1002/art.41156/abstract.) The sorted cells were then stimulated ex vivo and stained for IL‐17A and IFNγ (Figures 5F and G). After normalizing for total cytokine expression, we observed that the CD69+CD103+CD8+ T cell population contained the highest frequency of IL‐17A+ cells, followed by cells expressing either CD69 or CD103 only, while CD69−CD103−CD8+ T cells contained minimal IL‐17A+ cells (Figure 5H). In contrast, frequencies of IFNγ+ cells were distributed more equally among the 4 sorted cell subsets. We also found a significant correlation between the presence of IL‐17A+CD8+ T cells and CD69+CD103+ Trm cells in the SF, further supporting a relationship between these 2 cell populations (Supplementary Figure 5, available on the *Arthritis & Rheumatology* web site at http://onlinelibrary.wiley.com/doi/10.1002/art.41156/abstract). Taken together, these data indicate that synovial Trm cells are enriched for IL‐17A expression and that Tc17 cells form part of the Trm cell pool in the inflamed synovial joints in PsA.

#### Synovial Tc17 cells are polyfunctional inflammatory cells characterized by high expression of CXCR6

**Figure 6:**
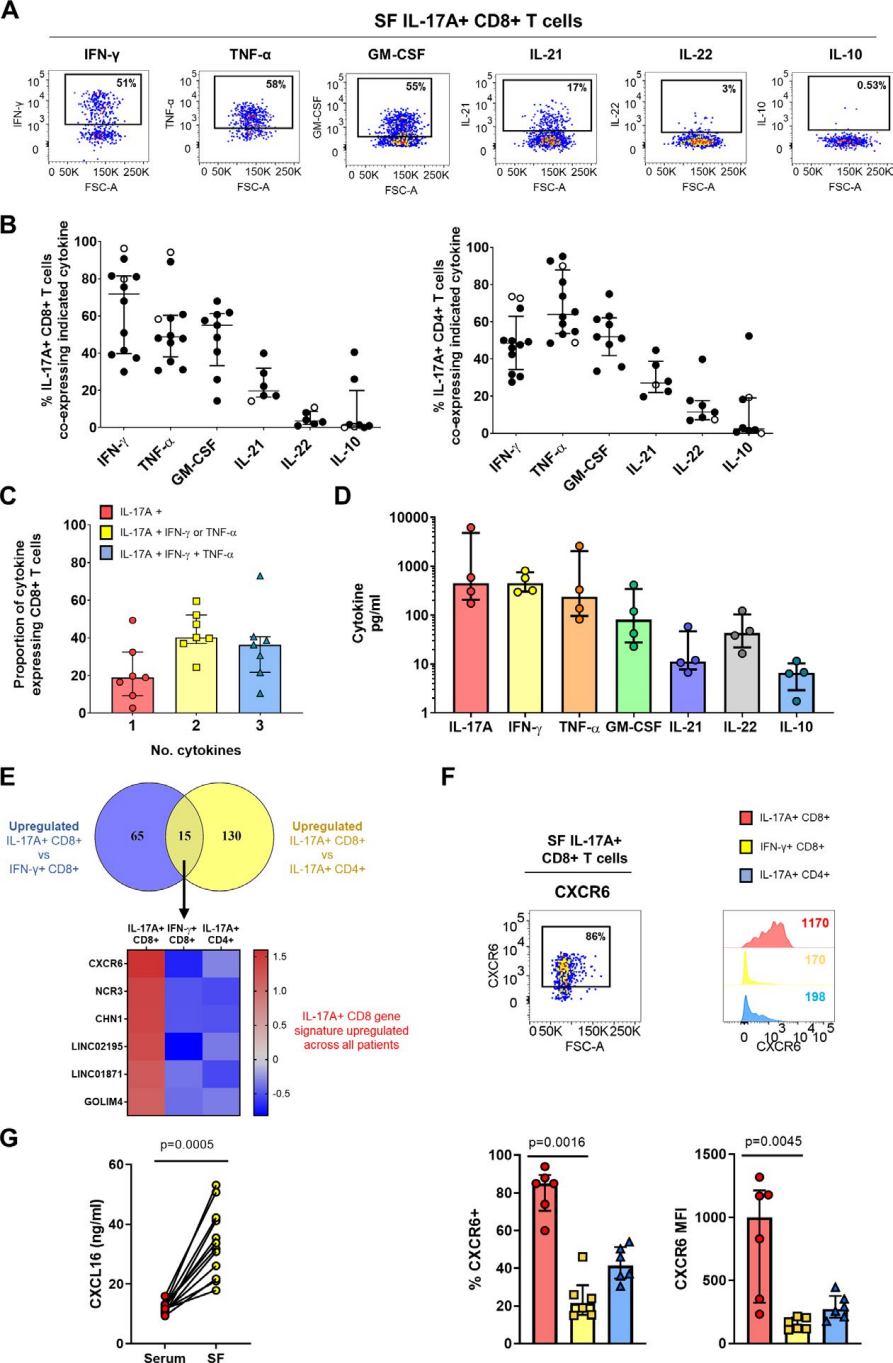
Synovial Tc17 cells are polyfunctional proinflammatory cells characterized by high expression of CXCR6. **A** and **B**, Representative staining (**A**) and cumulative data (**B**) showing the frequencies of IL‐17A+CD8+ (left) and IL‐17A+CD4+ (right) T cells expressing IFNγ (n = 12), tumor necrosis factor (TNF; n = 12), granulocyte–macrophage colony‐stimulating factor (GM‐CSF; n = 9), IL‐21 (n = 6), IL‐22 (n = 6), and IL‐10 (n = 8) in synovial fluid mononuclear cells (SFMCs) from patients with PsA (solid symbols) or other types of spondyloarthritis (open symbols). **C**, Proportion of IL‐17A+CD8+ T cells expressing IL‐17A alone, IL‐17A plus IFNγ or TNF, and IL‐17A plus IFNγ and TNF. **D**, IL‐17A, IFNγ, TNF, GM‐CSF, IL‐21, IL‐22, and IL‐10 secretion by sorted synovial IL‐17A+CD8+ T cells (n = 4). **E**, Top, Venn diagram showing the number of significantly up‐regulated genes (*P* < 0.01) in IL‐17A+CD8+ T cells versus IFNγ+CD8+ T cells and in IL‐17A+CD8+ T cells versus IL‐17A+CD4+ T cells. Bottom, Heatmap showing genes that were consistently up‐regulated in all patients. **F**, Representative dot plot and histograms (top) and cumulative data (bottom) showing the percentage of CXCR6+ cells and CXCR6 expression levels in PsA synovial IL‐17A+CD8+ T cells, IFNγ+CD8+ T cells, and IL‐17A+CD4+T cells (n = 6). *P* values were determined by Friedman's multiple comparisons test. **G**, CXCL16 levels in paired PsA serum and SF samples (n = 12) as measured by enzyme‐linked immunosorbent assay. *P* value was determined by Wilcoxon's matched pairs signed rank test. In **C** and **D**, bars show the median and interquartile range. MFI = mean fluorescence intensity (see Figure 2 for other definitions). Color figure can be viewed in the online issue, which is available at http://onlinelibrary.wiley.com/doi/10.1002/art.41156/abstract.

Finally, we sought to determine the functional potential of synovial Tc17 cells. For this, we assessed the coexpression and secretion of proinflammatory and antiinflammatory cytokines by flow cytometry and Luminex assay. A substantial proportion of synovial Tc17 cells coexpressed proinflammatory IFNγ, TNF, and granulocyte–macrophage colony‐stimulating factor (GM‐CSF), while IL‐21 was coexpressed by <20% of Tc17 cells. IL‐22 and antiinflammatory IL‐10 were found to be either absent or expressed by only a small proportion of Tc17 cells (Figures 6A and B). IL‐17F was found to be coexpressed by only a small proportion of Tc17 cells (Supplementary Figure 6B, available on the *Arthritis & Rheumatology* web site at http://onlinelibrary.wiley.com/doi/10.1002/art.41156/abstract). The cytokine coexpression profile of synovial Tc17 cells closely resembled the cytokine profile of synovial Th17 cells (Figure 6B), while this profile was not shared by synovial IL‐17A−CD8+ or IFNγ+CD8+ T cell subsets (Supplementary Figure 6A). Overall, synovial Tc17 cells displayed a polyfunctional cytokine profile with a sizable proportion of cells (median 36%) expressing IL‐17A, IFNγ, and TNF concomitantly (Figure 6C). This concomitant cytokine production was also observed in the Luminex analysis: sorted IL‐17A+CD8+ T cells from the SF of patients with PsA produced IL‐17A, IFNγ, TNF, GM‐CSF, and IL‐22, with low levels of IL‐21 and IL‐10, during a 24‐hour culture (Figure 6D). (The gating strategy is shown in Supplementary Figure 7, available on the *Arthritis & Rheumatology* web site at http://onlinelibrary.wiley.com/doi/10.1002/art.41156/abstract.)

Finally, we investigated whether Tc17 cells from the inflamed PsA joint express a unique set of markers. For this analysis, we compared the transcriptional profile of synovial Tc17 cells to the profiles of both synovial Tc1 and Th17 cells, to determine which markers are uniquely up‐regulated in synovial Tc17 cells. Bioinformatics analysis revealed that 15 genes were up‐regulated in both comparisons, suggesting that synovial Tc17 cells have a small unique transcriptional profile in comparison to synovial Tc1 and Th17 cells (Figure 6E). Of these 15 genes, 6 were found to be consistently up‐regulated in all 3 patients: *CXCR6*,* NCR3*,* CHN1*,* LINC02195*,* LINC01871*, and *GOLIM4*. Since CXCR6 is part of the Trm gene signature 16, we validated CXCR6 at the protein level and found that PsA synovial Tc17 cells indeed expressed CXCR6 at the highest level and contained the highest proportion of CXCR6‐expressing cells as compared to their synovial Tc1 and Th17 counterparts (Figure 6F). In addition, levels of CXCL16, the ligand for CXCR6, were significantly increased in PsA SF versus paired serum samples (Figure 6G).

## Discussion

We previously described an enrichment of IL‐17A+CD4− (the majority of which were CD8+) T cells in the SF, compared to the PB, in patients with PsA 10. In this study we showed that frequencies of IL‐17A+CD8+ T cells were also increased in the SF of patients with other types of SpA, and confirmed that these cells were not increased in patients with RA 10. These findings add to the growing evidence that CD8+ T cells and the IL‐17/IL‐23 axis are relevant to the immunopathogenesis of PsA/SpA 2. We demonstrated that the vast majority of PsA synovial Tc17 cells are memory cells, indicated by a CD45RA−PD‐1+HLA–DR+ profile, as well as TCRαβ bearing, suggesting that these cells are MHC class I restricted and antigen‐experienced.

Previous studies demonstrated oligoclonal expansion of joint‐derived bulk T cells in PsA 21, 22, 23. Our TCRβ sequencing of synovial Tc17 cells, although limited in sample size, showed that while some T cell clones occupied >5% of the total TCR repertoire, the majority of Tc17 cells, as well as of the bulk memory CD8+ T cells and Tc1 cells, had a polyclonal TCR repertoire. One reason for the differences observed between the previous studies and our own could be patient disease activity. A previous study investigating the clonality of psoriatic skin–derived CD8+ T cells showed that the TCR repertoire is polyclonal in active disease, while in resolved disease only a few dominant clones persist 28. We obtained SF effusions from joints with active inflammation, which could explain the polyclonal repertoire observed. It would be of interest to investigate whether a polyclonal repertoire is also observed in joints in which PsA has resolved; however, this would require a synovial biopsy study design since noninflamed joints rarely contain sufficient SF for aspiration.

A notable observation in our analysis was the extensive sharing of T cell clones between the synovial Tc17 and Tc1 populations. Our RNA‐Seq analysis also showed significant overlap in transcriptional profile between Tc17 and Tc1 cells. A possible explanation for these findings is that synovial Tc17 and Tc1 cells may have shared ancestry, which raises the question of plasticity between these subsets. Adoptive transfer of in vitro generated Tc17 cells into recipient mice showed that Tc17 cells can switch to an IL‐17A–negative Tc1 profile 29, 30, 31. A direct demonstration of this phenomenon came from an elegant IL‐17A fate‐mapping study using reporter donor IL‐17^Cre^Rosa26^eYFP^ 13. That study showed that Tc17 cells developed early after allogeneic stem cell transplantation in both lymphoid tissue and graft‐versus‐host disease target organs. However, their production of IL‐17A was transient, while IFNγ production was largely maintained, a process defined by the surrounding cytokine milieu. A similar capacity to transition to IFNγ production and Th1 phenotype had previously been shown for mouse Th17 cells 32. These fate‐mapping data, taken together with our findings on shared TCR and transcriptional profiles and the observation that a high frequency of synovial Tc17 cells express IFNγ, suggest that synovial Tc17 cells can transition to a Tc1‐like cytokine profile, as was previously suggested for Th17 and Th1 cells in the joints of patients with JIA 33. If this scenario is indeed the case, then one implication of this finding would be that the percentage of IL‐17A+CD8+ T cells detected by flow cytometry may underrepresent the contribution that Tc17 cells make or have made to the synovial T cell compartment.

In addition to Tc17/Tc1 overlap, we observed a phenotypic and molecular overlap between Tc17 and Th17 cells in PsA, as evaluated by RNA‐Seq, qRT‐PCR, and flow cytometry. Two markers typically expressed by Th17 cells, CCR6 and CD161 34, 35, 36, are coexpressed by a large proportion of synovial Tc17 cells. Furthermore, expression of *IL23R* by synovial Tc17 cells indicates that IL‐23 may be involved in the generation or maintenance of these cells, while elevated *RORC* transcript expression suggests that RORγt may at least play a part in IL‐17A regulation in these cells. These data indicate that Tc17 cells may be regulated by similar pathways as Th17 cells.

The relatively limited sample sizes in the TCR and RNA‐Seq analysis could be considered a limitation of our study. However, given the challenges in obtaining sufficient numbers from small populations of immune cells, this number of samples (n = 3) is not uncommon in studies of human tissue‐derived cells. Additionally, many of our RNA‐Seq results were independently validated at the protein or RNA level. A further limitation of our study is that we did not have access to synovial tissue samples from patients with PsA. Nonetheless, the work presented here provides an important basis for future studies aimed at comparing the phenotype and molecular profile of SF‐ and synovial tissue–derived Tc17 cells.

A key novel finding of our study is that synovial Tc17 cells are part of the Trm cell compartment and express molecules that prevent egress from the inflamed tissue into the blood. Trm cells are rapidly emerging as potential contributors to inflammation in several immune‐mediated inflammatory diseases 17, 37. To our knowledge, this is the first study to show that Tc17 cells form part of the synovial Trm pool, and only the second description of Trm‐like cells in the context of human immune‐mediated arthritis 11. Our data also show that a high proportion of synovial Tc17 cells express markers typically associated with homing to the skin or gut. This finding could indicate that synovial Tc17 cells have tropism for these tissues as well as for the synovial compartment, and indeed, the presence of Tc17 cells has been described in both the skin and the gut 24, 25, 27. An alternative explanation could be that synovial Tc17 cells expressing these markers have enhanced adherence to the surrounding joint tissue, as both CLA and CD49a ligands (E‐selectin and type IV collagen, respectively) are present in the synovial tissue 38, 39. β7 integrin, which is expressed by a large proportion of synovial Tc17 cells, can form a heterodimer with the Trm cell marker CD103 (αE integrin), which is expressed by Tc17 cells. The product, αEβ7 integrin, exclusively binds E‐cadherin, a structural protein expressed in the synovi‐al joint and fluid in patients with inflammatory arthritis 39, 40. Interaction of synovial Tc17 cells with the surrounding tissue and extracellular matrix in the fluid combined with lack of response to exit cues (e.g., S1P1) could represent one way in which Tc17 cells are retained in the synovial joint and contribute to perpetuation or re‐initiation of inflammation.

Residence of synovial Tc17 cells in the inflamed tissue may be further enhanced by their high expression of CXCR6, a marker of Trm cells 16, in combination with the increased levels of CXCL16 in the PsA synovial joint. CXCL16 has chemotactic and angiogenic properties and can be produced as a soluble mediator or as a transmembrane‐bound chemokine by monocytes, macrophages, and dendritic cells. Evidence of increased CXCL16 and CXCR6 expression at the site of inflammation was reported previously in the context of RA and psoriasis, and CXCL16 was shown to enhance recruitment of inflamed tissue‐derived CXCR6‐expressing T cells 41, 42, 43. Furthermore, CXCL16 blockade or CXCR6 deficiency led to reduced arthritis scores and lower IFNγ/IL‐17 production in an experimental model of arthritis 42, 44. Taken together, these data suggest that the increased CXCR6 expression on synovial Tc17 cells may contribute to their recruitment and persistence in the inflamed PsA joint.

Functionally, our data indicate that synovial Tc17 cells are polyfunctional and actively secrete several proinflammatory cytokines (IFNγ, TNF, GM‐CSF, IL‐21, and IL‐22) in parallel with IL‐17A, but little IL‐10 or IL‐17F. IFNγ, TNF, and GM‐CSF have all been shown to act synergistically with IL‐17A to promote inflammation. In a recent study by Wade et al, polyfunctional CD8+ T cells were also found to be enriched in the PsA synovium, although enrichment was not observed for single cytokine–producing T cells, including IL‐17A+CD8+ T cells 45. Concordant with their proinflammatory cytokine production, Tc17 cells coexpressed cytolytic molecules granzyme A and granzyme B at levels comparable to those in sy‐novial Tc1 cells. However, Tc17 cells lacked significant expression of other cytolytic machinery (perforin and LAMP‐1). Most studies to date in both mice and humans have shown that Tc17 cells lack cytolytic function 29, 31, 46, 47, although some evidence for cytotoxic function has been reported 24, 48. An interesting alternative interpretation of our data is that the secretion of granzymes is not cytolytic but leads to extracellular matrix degradation or promotion of inflammation 49, 50. Taken together, these data show that Tc17 cells are armed with an array of proinflammatory mediators, which could act directly on surrounding cells to promote inflammation in the synovial joint.

In summary, our findings reveal that synovial Tc17 cells from the PsA joint bear hallmarks of Trm cells, and express high levels of CXCR6, which may enhance retention of these cells in the inflamed joint. Our analysis of the transcriptional profile and TCR repertoire of these cells highlights several commonalities between Tc17 and Tc1 cells in the PsA joint. Combined with the observed polyfunctional proinflammatory mediator production, we hypothesize that Tc17 cells exert heterogenous effector responses that contribute to the initiation and persistence of PsA.

## Author Contributions

All authors were involved in drafting the article or revising it critically for important intellectual content, and all authors approved the final version to be published. Dr. Taams had full access to all of the data in the study and takes responsibility for the integrity of the data and the accuracy of the data analysis.

### Study conception and design

Steel, Kirkham, Taams.

### Acquisition of data

Steel, Srenathan, Durham, Wu, Ryan, Hughes, Kirkham, Taams.

### Analysis and interpretation of data

Steel, Srenathan, Ridley, Durham, Wu, Ryan, Chan, Kirkham, Taams.

## Role of The Study Sponsor

Novartis Pharma AG had no role in the study design or in the collection, analysis, or interpretation of the data, the writing of the manuscript, or the decision to submit the manuscript for publication. Publication of this article was not contingent upon approval by Novartis Pharma AG.
