# Supplementary figures and images for: Polyfunctional, Proinflammatory, Tissue‐Resident Memory Phenotype and Function of Synovial Interleukin‐17A+CD8+ T Cells in Psoriatic Arthritis
Source: Arthritis Rheumatol. 2020 Feb 4;72(3):435–47. doi: 10.1002/art.41156 (PMC7065207; doi:10.1002/art.41156)

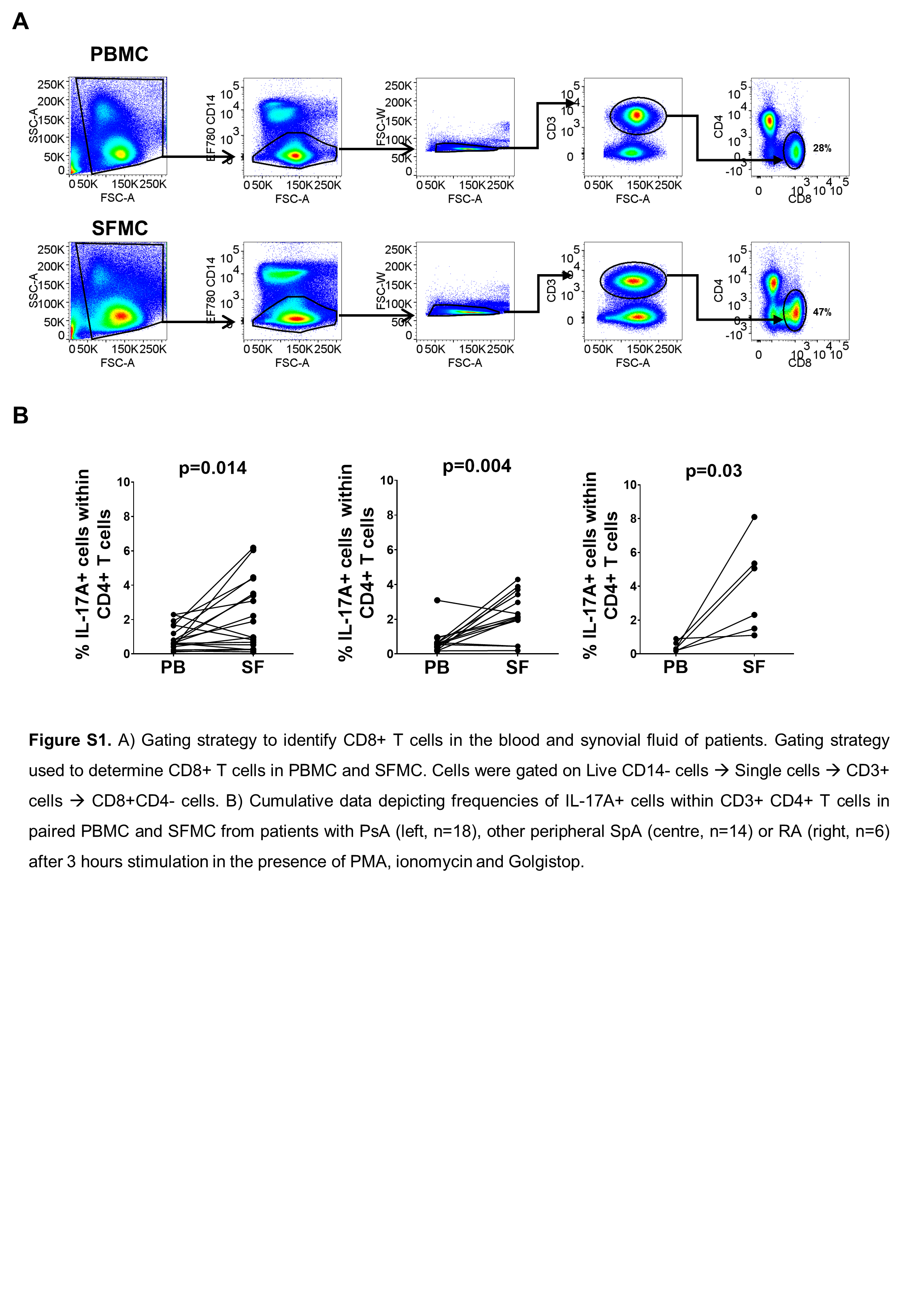

Supplement: Supplementary file 1 [file ART-72-435-s001.TIF]

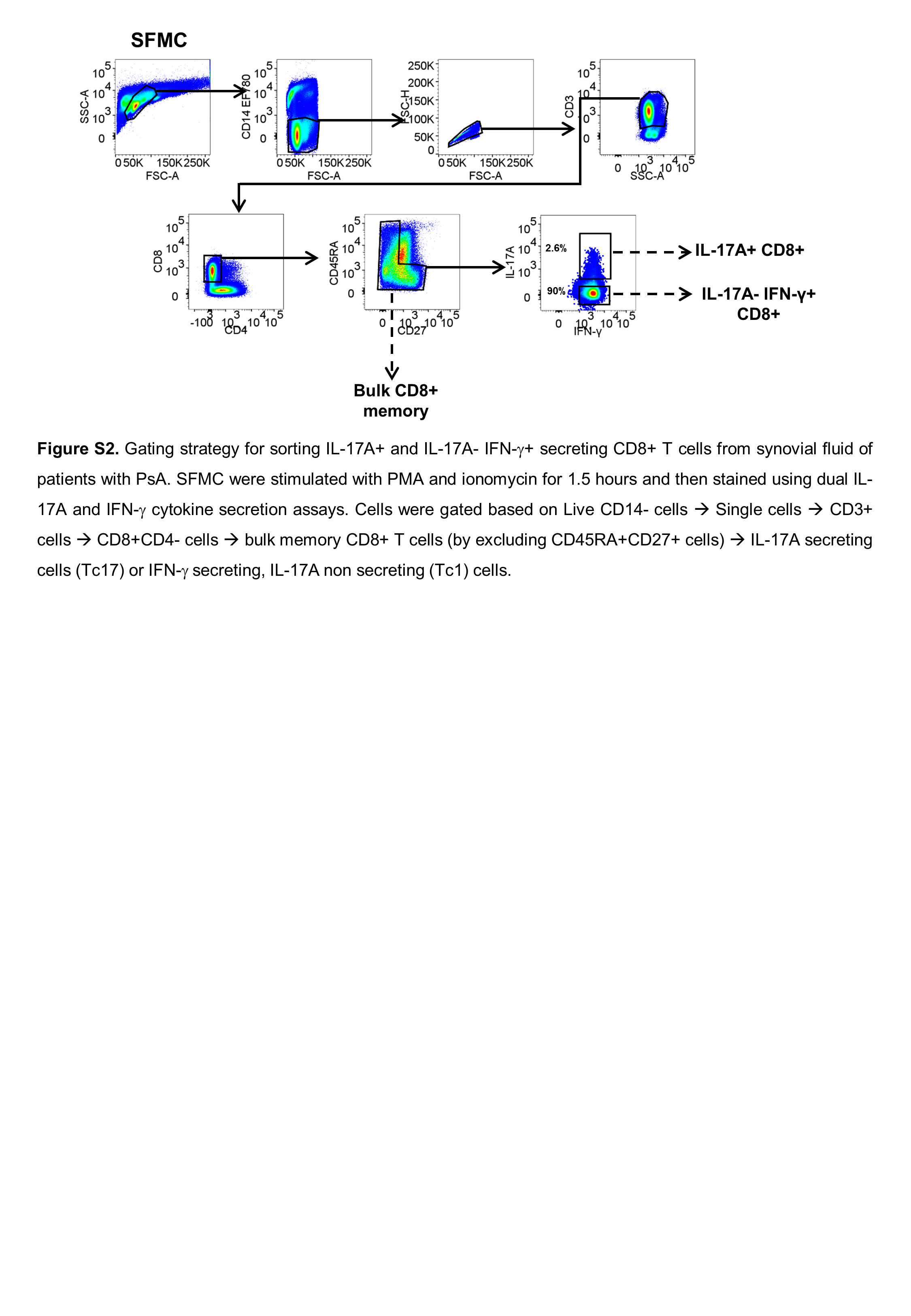

Supplement: Supplementary file 2 [file ART-72-435-s002.TIF]

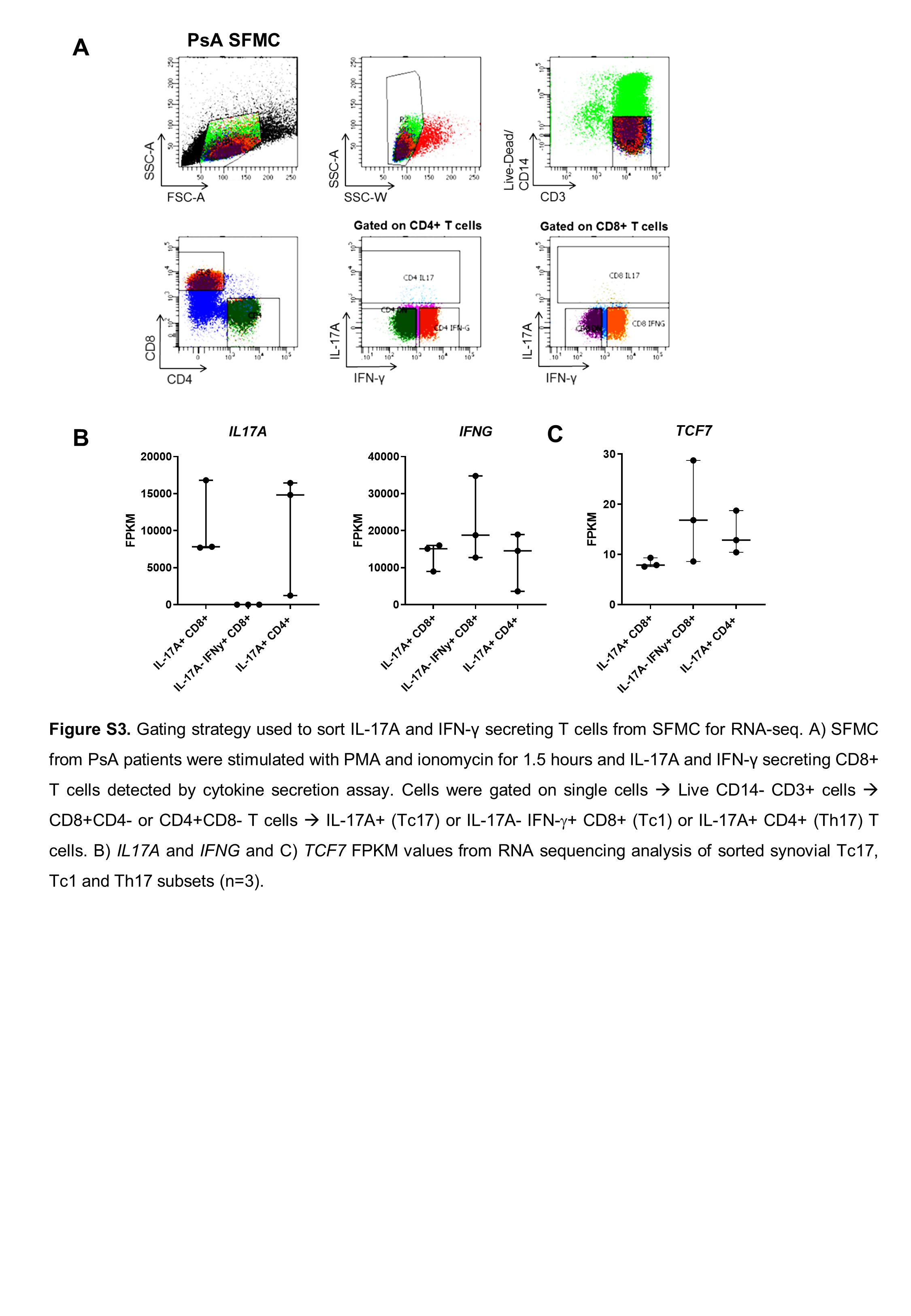

Supplement: Supplementary file 3 [file ART-72-435-s003.TIF]

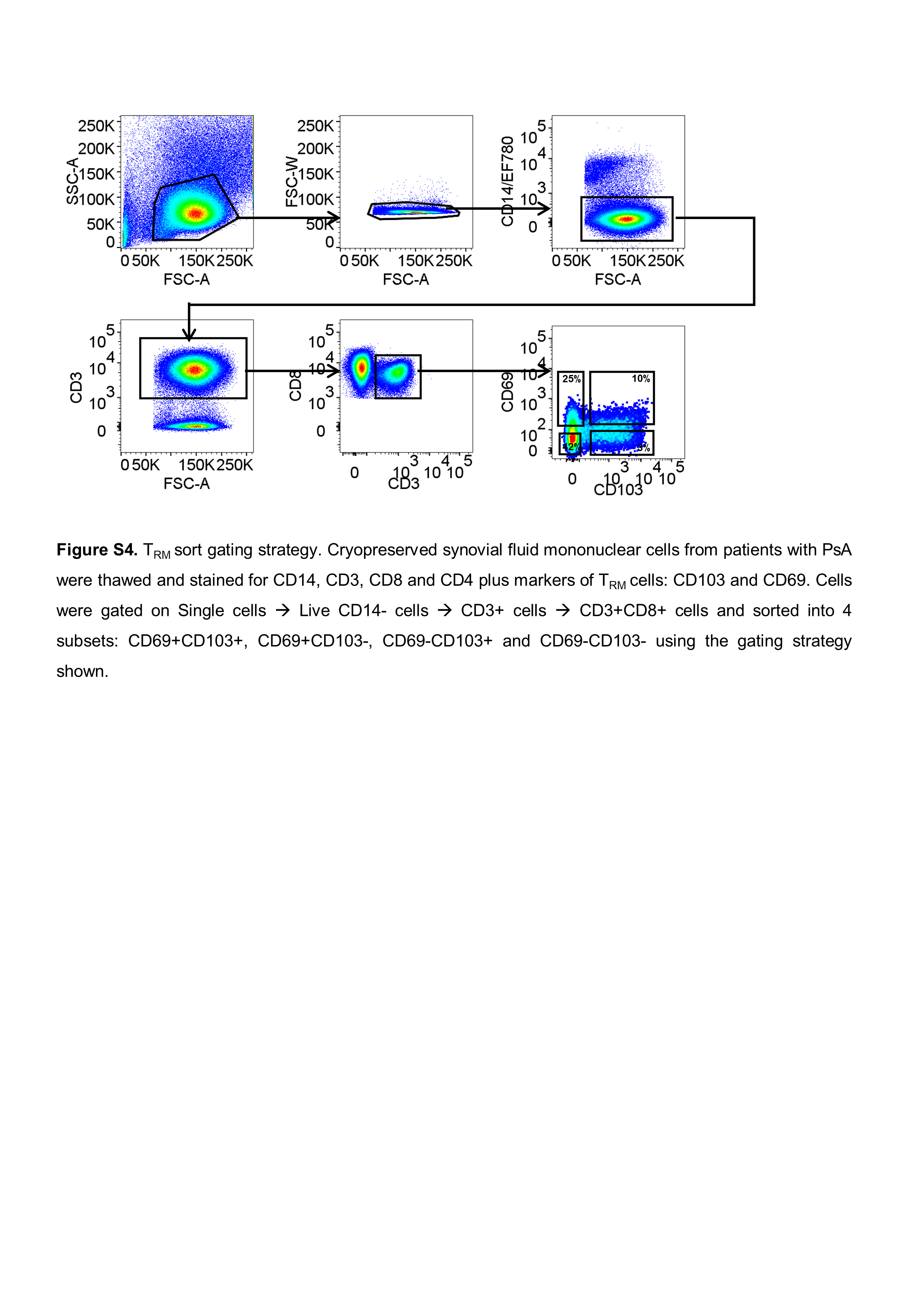

Supplement: Supplementary file 4 [file ART-72-435-s004.TIF]

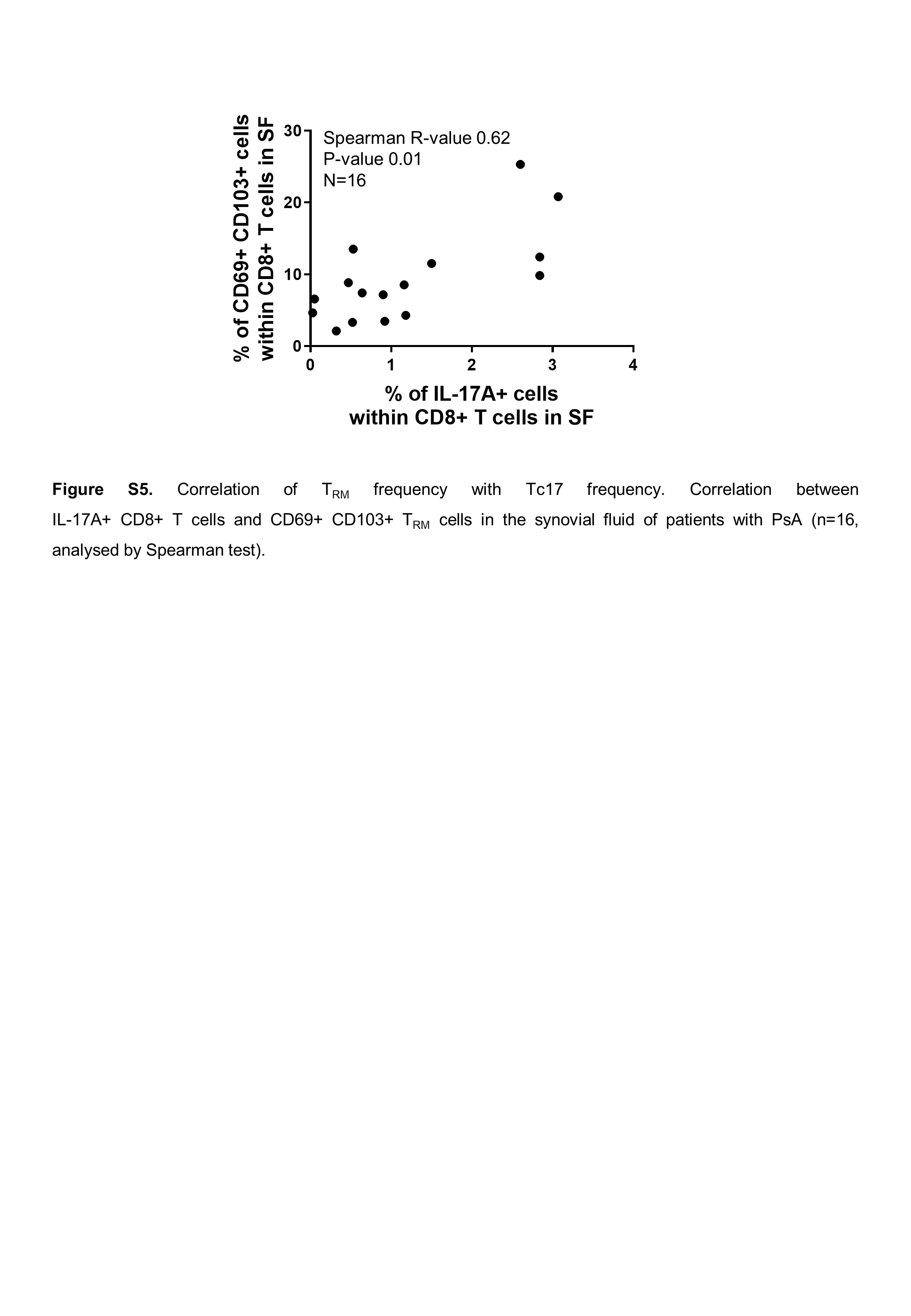

Supplement: Supplementary file 5 [file ART-72-435-s005.TIF]

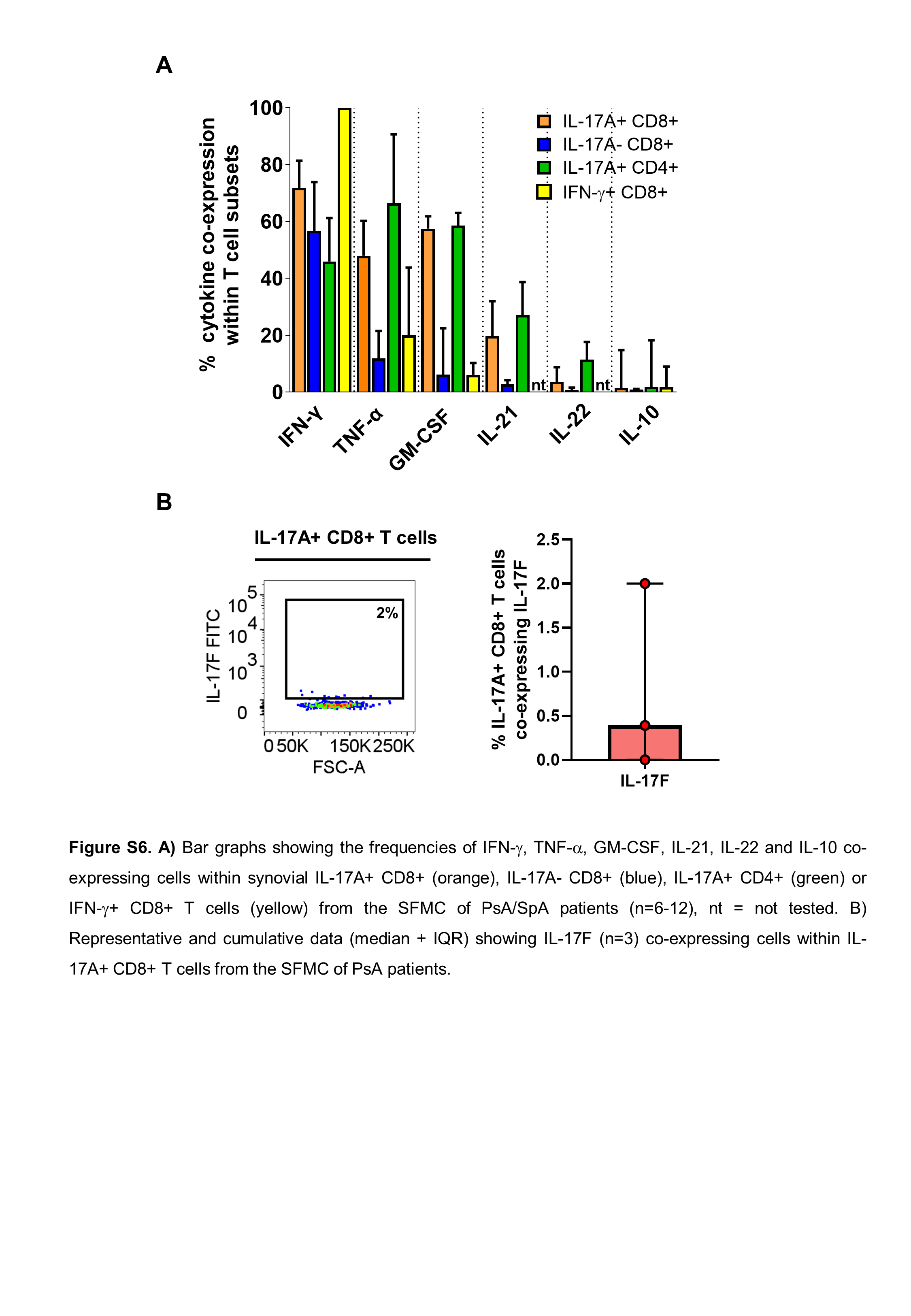

Supplement: Supplementary file 6 [file ART-72-435-s006.TIF]

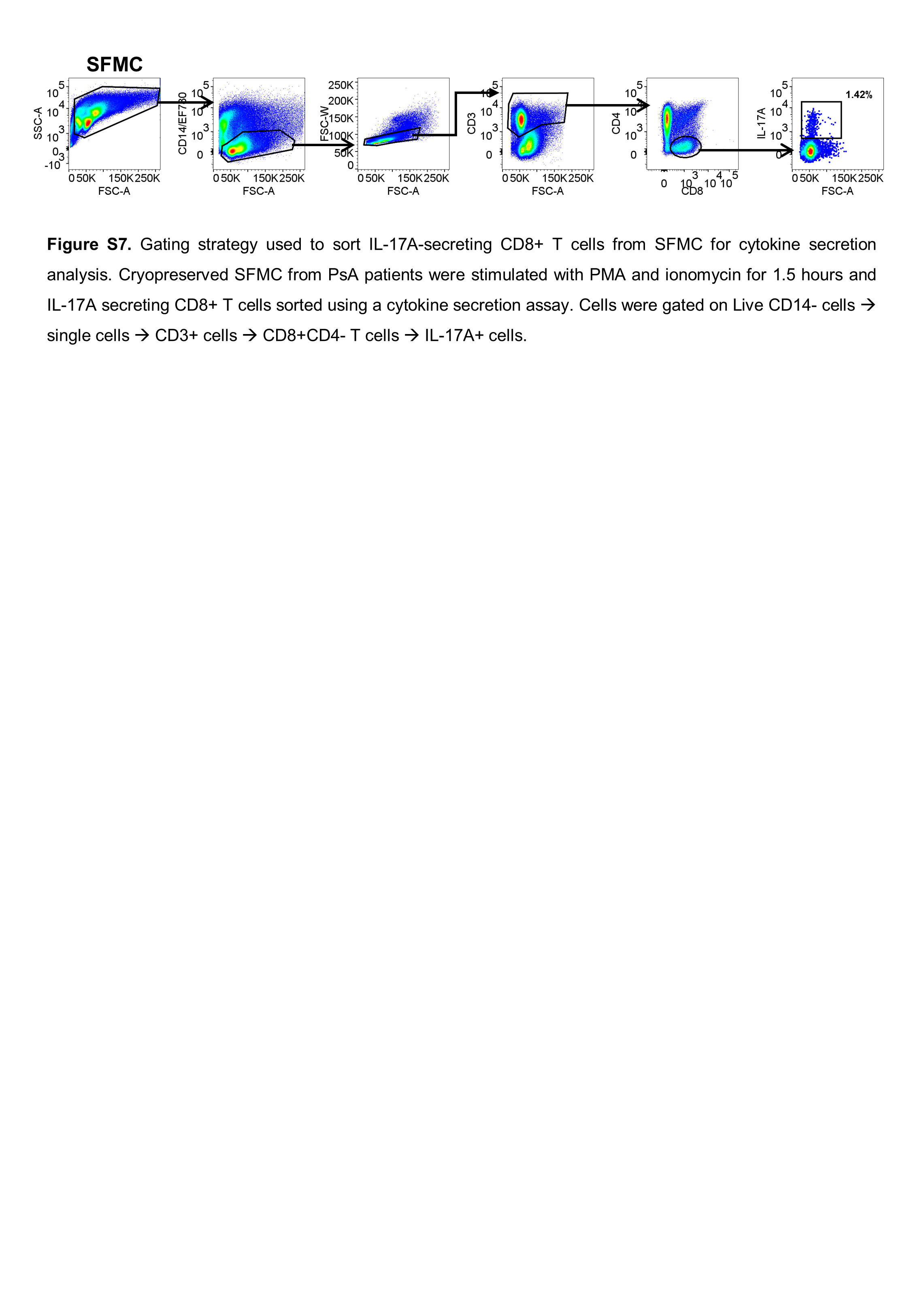

Supplement: Supplementary file 7 [file ART-72-435-s007.TIF]

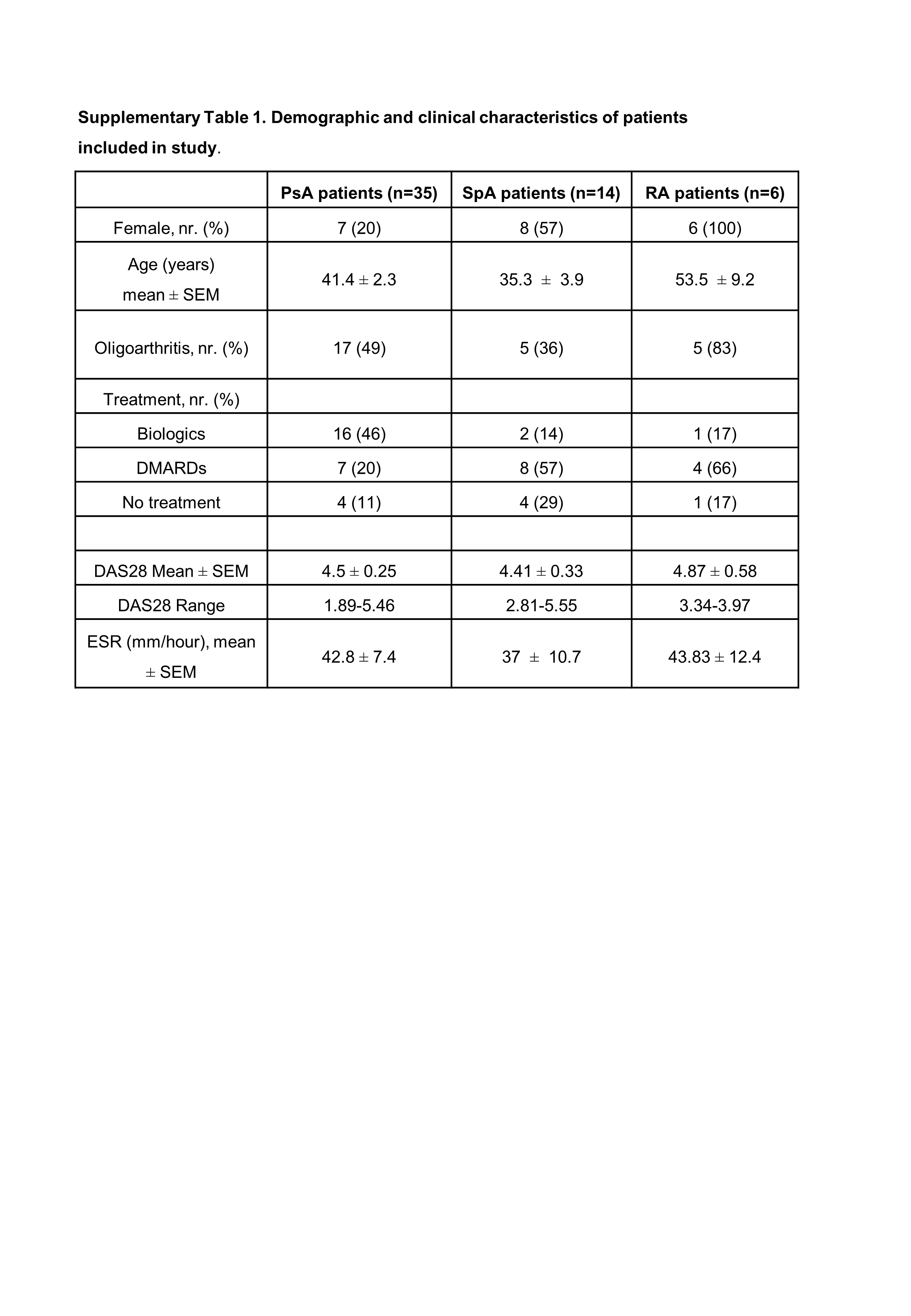

Supplement: Supplementary file 8 [file ART-72-435-s008.TIF]

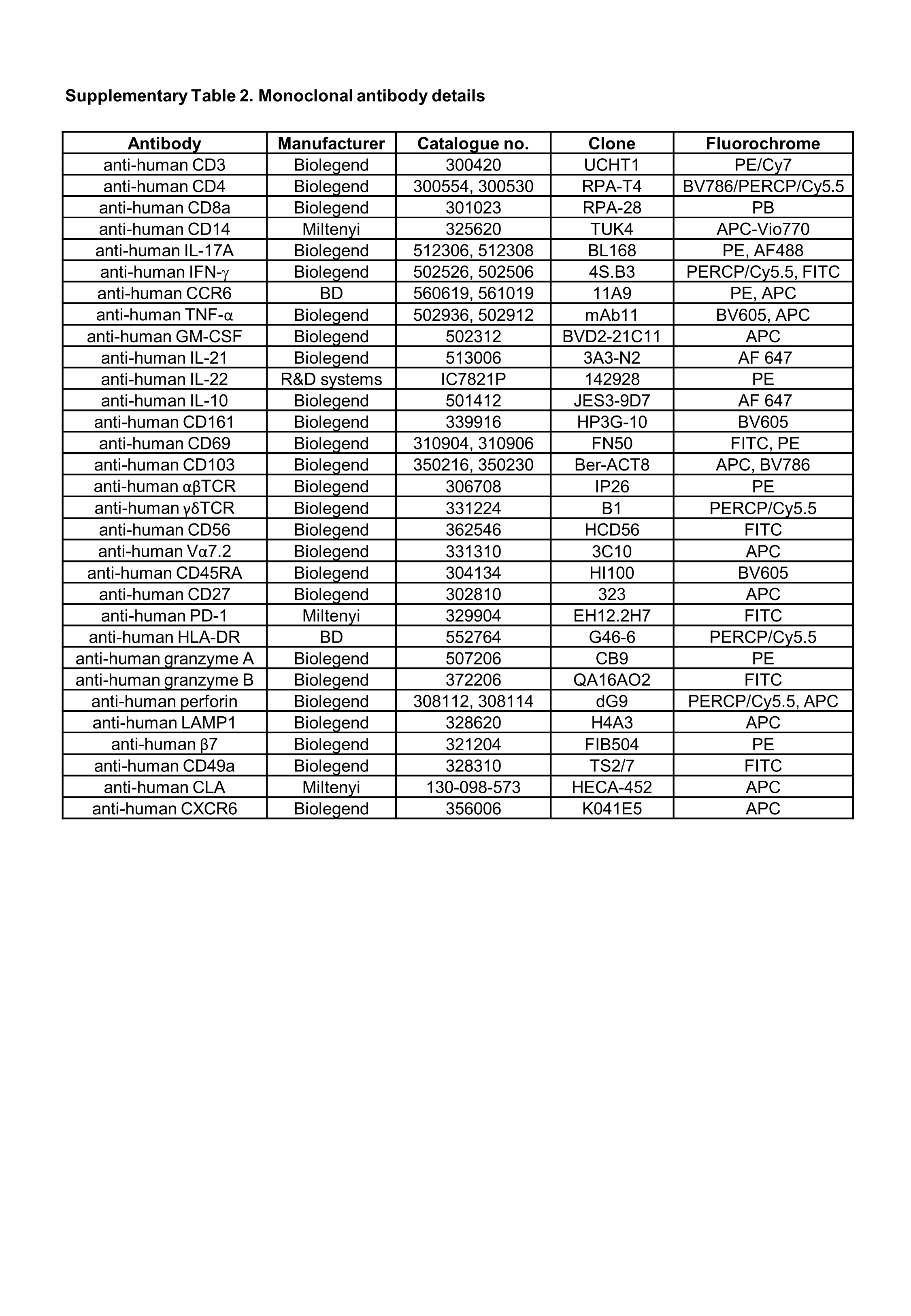

Supplement: Supplementary file 9 [file ART-72-435-s009.TIF]

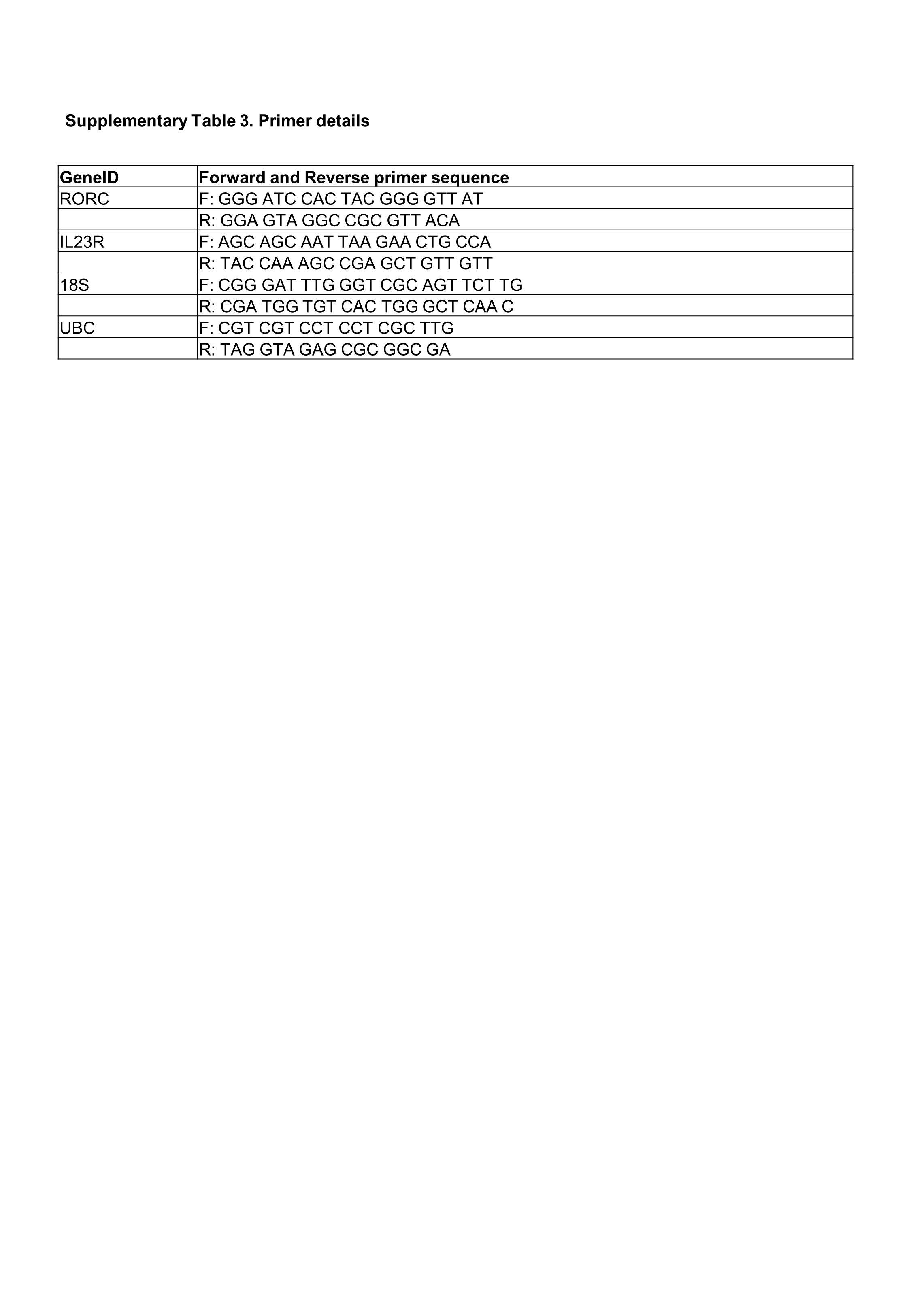

Supplement: Supplementary file 10 [file ART-72-435-s010.TIF]
